# An Open Localization and Local Communication Embodied Sensor

**DOI:** 10.3390/s8117545

**Published:** 2008-11-25

**Authors:** Álvaro Gutiérrez, Alexandre Campo, Marco Dorigo, Daniel Amor, Luis Magdalena, Monasterio-Huelin Félix

**Affiliations:** 1 ETSI Telecomunicación, Universidad Politécnica de Madrid, Avd. Complutense S/N, 28040 Madrid, Spain; 2 IRIDIA, CoDE, Université Libre de Bruxelles, 50, Av. F. Roosevelt, CP 194/6, Brussels, Belgium; 3 RBZ Robot Design S.L., Avd. Vía Láctea S/N, Local 30, C.M.E. San Fernando de Henares, 28830 Madrid, Spain; 4 European Centre for Soft Computing, C. Gonzalo Gutiérrez Quirós S/N, 33600 Asturias, Spain E-mails: acampo@iridia.ulb.ac.be (A. C.), mdorigo@ulb.ac.be (M. D.), daniel@rbz.es (D. A.), luis.magdalena@softcomputing.es (L. M.), felix.monasteriohuelin@upm.es (F. M.-H.)

**Keywords:** Localization, local communication, range and bearing, embodied communication

## Abstract

In this paper we describe a localization and local communication system which allows situated agents to communicate locally, obtaining at the same time both the range and the bearing of the emitter without the need of any centralized control or any external reference. The system relies on infrared communications with frequency modulation and is composed of two interconnected modules for data and power measurement. Thanks to the open hardware license under which it is released, the research community can easily replicate the system at a low cost and/or adapt it for applications in sensor networks and in robotics.

## 1. Introduction

Sensor networks consist of spatially distributed autonomous nodes which collectively monitor the environment and coordinate to resolve specific tasks [1, 2]. A node is typically equipped with several sensors, a wireless communication device, a controller and an energy source. Each node has limited capabilities but the coordination of all the nodes of a network enables them to complete a given task. Sensor networks are currently used in many civilian application areas such as environment monitoring [3], security systems [4], home automation [5], traffic control [6], medical applications [7] or efficient energy consumption [8]. To carry out these tasks, the nodes must coordinate their activities by exchanging information through a communication system.

Communication can be separated in two categories, namely abstract or situated. Abstract communication [9] refers to communication protocols in which only the content of the message carries a meaning and the physical properties of the signal that transports the message do not have any semantic. Sensor network applications as those mentioned above make use of abstract communication in static networks where the nodes have fixed locations. Situated communication refers to interactions in which the physical instantiation of the message contributes to define its semantics [10].

The majority of communication systems implemented in sensor networks make extensive use of abstract communication using radio devices. In these systems, nodes are missing useful information such as the location of the emitter of a message. This problem has been addressed in works such as [11, 12], but the nodes must usually be programmed with a model of the environment or the physical location and topology of the network. For example, in wireless mobile applications, nodes commonly calibrate and triangulate their positions according to a model obtained in the design process [13, 14]; in home automation sensor networks [15], a map of the rooms and of the nodes locations is predefined and programmed on the network, while in security mobile systems a map of the environment is offered to the agents which then apply fusion techniques for locating themselves and their teammates [16]. These implementations are functional in a given environment for which they must be configured using appropriate models. When the network is moved to a new environment, the modelling step must be repeated.

Following modern artificial intelligence approaches [17, 18], new functionalities and autonomies are to be given to the networks, which should discover the environment and self-organize their topology. Situated communication provides a simple and elegant solution to these approaches, where the nodes (mobile or static) can identify the location of their neighbors relatively to their body and situation [19, 20]. Nodes that receive a message also infer the relative position (both range and bearing) of the emitter. Therefore, when a node is moved from one place to another, the rest of the network nodes are able to recognize its new location.

Localization and communication systems have been designed and studied in several previous works. Some implementations based on GPS [21] or on triangulation based on external fixed devices [13], make use of absolute localization systems which provide the nodes with positional information in a global coordinate framework. However, there are situations in which it is not possible to use absolute localization. This is the case, for example, when neither is possible to receive GPS signals, nor to augment the environment with the devices necessary for triangulation. Other implementations based on relative localization systems make use of an extensive variety of technologies (e.g. radio, ultrasound, infrared). In [22] is proposed a Bluetooth based localization solution that, although having a good performance, requires the transmitting device to remain stationary during the period of time (at least five minutes) during which it is inquired by the network. In [23] an algorithm is derived from the position of ZigBee devices by averaging the coordinates of known reference points. Implementations based on radio devices commonly use external devices as landmarks to achieve situated communication. Nonetheless, using radio communications for relative localization without any external fixed beacon could be achieved. However, to accomplish the same resolution as with ultrasonic or infrared technologies it is necessary to use a high frequency system combined with the use of directional antennas. This implementation results in a too big and expensive solution for being implemented in small size nodes. Finally, radio implementations are less energy efficient than implementations based on ultrasound or infrared [24, 25].

An ultrasonic localization system is described in [26], but it suffers from accuracy problems. On the other hand, [27] accomplished a very accurate relative positioning using ultrasound, but tests were never performed with more than two nodes. The use of ultrasound suffers from echo effects and interference that reduce the performance when more nodes are introduced in the system. Another problem is that the aperture of the ultrasonic emitters is not narrow enough to achieve a good directionality.

Finally, infrared sensors have been previously used for relative positioning systems taking advantage of their directionality. In [28, 29] an infrared localization system based on a narrow-band FM demodulator is implemented. The system achieves a date rate up to 20 Hz and the range of the module goes up to 310 cm with an average standard deviation of 6.10*^°^* in bearing and 10.23 cm in range. However, due to the radio frequency electronics employed, the board is difficult to miniaturize. In [30, 31] another infrared-based system is implemented achieving similar performances, but there is not enough information to replicate the system.

In this paper, we pursue a relative localization system for miniaturized nodes which does not make use of any external device. We are interested in exploiting a general, robust and self-localization mechanism. The localization mechanism should allow a receiver to extract accurate distance and angle information without the need of any external device. We also focus on the design of a low consumption and accu-rate system. For these reasons, in this work we present the implementation of situated communication using an open hardware board where limited range communication and localization of emitters by receivers are achieved using infrared signals. Infrared is chosen because of the high directionality of the signals emitted, the low aperture angle of the receivers, the inexpensive transducers and the low power requirements. The board has been designed for robotics tasks (*Epuck Range & Bearing* board) but can be adapted to many other applications such as home automation, weather stations, security systems, and so on. Due to the many potential board usages, we focus on the hardware system abstracted from any specific application. The board is able to receive data and at the same time extract the emitter's range and bearing from the communication. All the specifications of the board are available under open hardware license which makes the board easily reproducible at a low production cost of less than 250 euros.

The paper is organized as follows. Section 2. describes the range and bearing hardware. In Section 3., we provide detailed information on the data fusion model in order to get correct location information. Experimental results are presented in Section 4. Finally, Section 5. concludes the paper and suggests future developments.

## 2. Localization and Communication System

**Figure 1.**
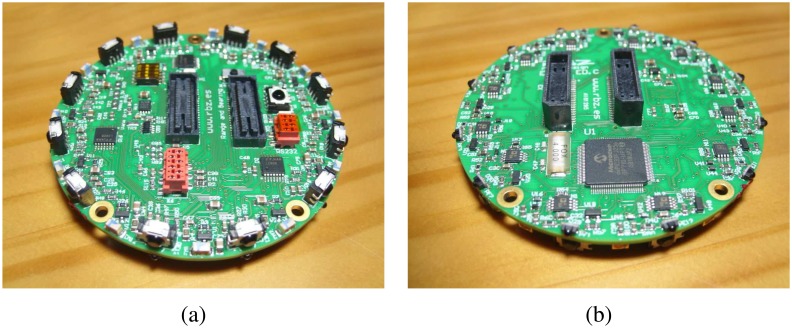
(a) Top and (b) bottom view of the range and bearing board.

**Figure 2.**
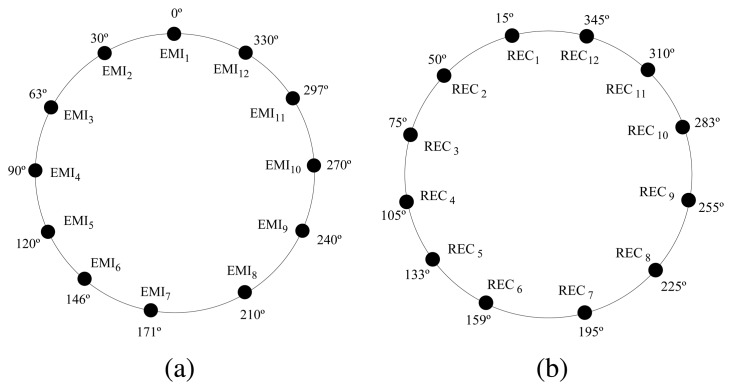
(a) Emitters and (b) receivers distribution around the perimeter of the board.

The designed range and bearing board (see Figure 1) is controlled by its own processor. Each board includes 12 sets of IR emission/reception modules. Each of these modules is equipped with one infrared emitting diode, one infrared modulated receiver and one infrared photodiode*. The modules, as shown in Figure 2, are nearly uniformly distributed on the perimeter of the board; so, the distance between them is approximately 30°.

For achieving local communication and localization, different modules are simultaneously controlled on the board. Once the board is powered, a sequence of timers starts. At the very beginning all the input/output ports and analog to digital ports are defined. The board has been designed to be the slave of a main processor system. Therefore, after the definition of the peripherals, a communication bus is started. Finally, the emission and reception modules are initialized. Communication is achieved through a modulated signal, offering robustness to light conditions.

Once the board is initialized, a pulse-width modulation (PWM) timer is initialized with a period of 1.09 *μs*. This timer creates the carrier of the emission module which will not be stopped until the board is powered down. A Manchester code is implemented to allow any data sent at a certain distance to be received with the same intensity by the receiver. The timer, which takes care of the modulated signal, interrupts each 100 *μs*. The implementation of the Manchester code allows a maximum data rate of 5 kbps. Each interruption of this timer takes the buffered data and sends it to the hardware gates for its transmission. Data for transmission is stored in a buffer correctly structured according to the hardware pinout. Three different types of transmission can be asked to the communication board:

- **All the sensors transmit the same data**: One instruction is sent to the board, along with the data to transmit.
- **Only some sensors transmit data**: One instruction for each sensor must be sent to the board. Data and sensor number must also be provided to the board. After all the sensors have been loaded, a “send” instruction must be sent to the board.
- **Different sensors transmit different data**: One instruction for each sensor must be sent to the board. Data and sensor number must also be provided to the board. After all the sensors have been loaded, a “send” instruction must be sent to the board.

Once a transmission order is sent by the master to the board, the communication module is in charge of decomposing the data for the different sensors with a preamble (2 bits), the data (8 bits) and a CRC (2 bits). If the master needs to transmit a flow of data, the communication module buffers all the messages one after the other, in a transparent manner for the transmission timer.

**Figure 3.**
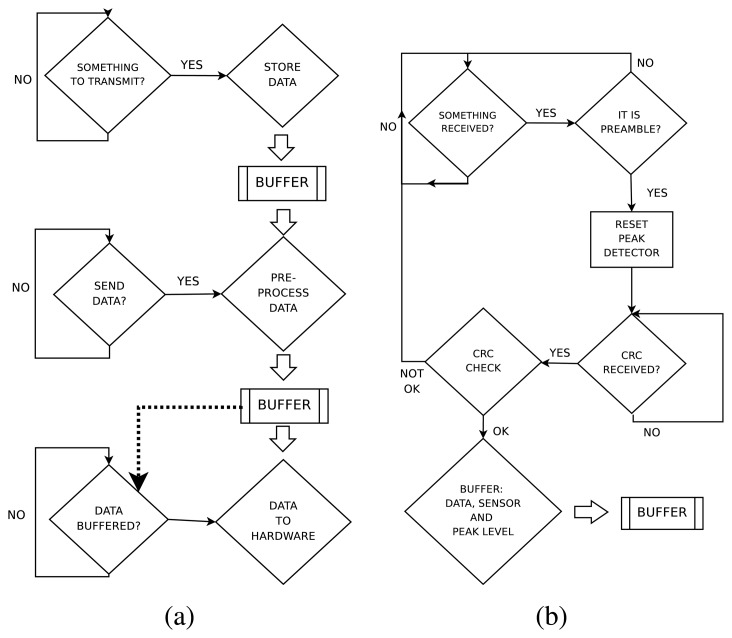
Block diagram of the (a) software emission module and (b) software reception module.

The reception software is continuously checking if a message arrives. Once the preamble of a frame is detected by an infrared modulated receiver, the board continues receiving the data and CRC while it is charging a peak detector through an infrared photodiode. If the frame has correctly arrived (checked by the CRC), the peak detector level is read and stored in a buffer. As the aperture of the receiving sensor is wide, it is likely that several sensors receive the same data at the same time. The information given by the different peak detectors is used to calculate the orientation and distance to the emitter. These two values are then stored in a buffer to be sent to the master board. Figure 3 shows a block diagram of the emission and reception software modules.

The board implements different mechanisms for signal interference and noise errors. In the first case, if two emitters are addressing the same receiver the transmission will be disrupted. Therefore, the CRC check will detect that the frame is not properly received: This will cause the frame to be discarded. Infrared noise comes mainly from light conditions in the environment. To deal with it, the board continuously measures the infrared signal in the environment. Once a frame is correctly received, the board subtracts the environment measure from the peak receptor and returns it as the frame signal intensity. This implementation allows the board to be moved from one place to another with different light conditions. Section 4. shows results for different light conditions.

For the correct understanding of the localization and communication system and its replication or modification possibilities, the forthcoming subsections detail the different hardware modules implemented in the board.

### 2.1. Power Supply Module

The board can be powered from 2.5 V to 6 V. Once the board is switched on, three isolated power lines are created: one for the digital system, one for the analog and the last one for the emission module. The three power lines are obtained from two different supplies.

**Figure 4.**
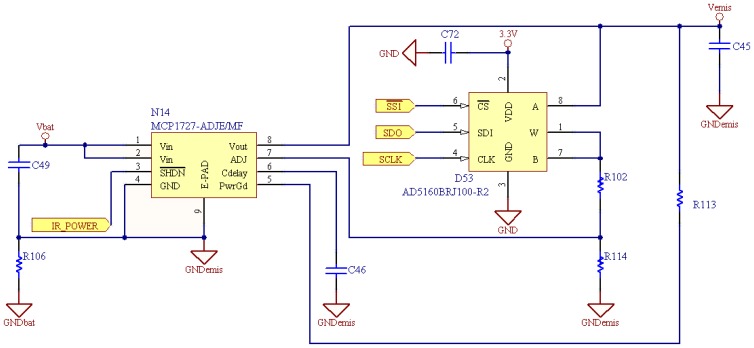
Schematic of the power supply in charge of the emission module.

The first power supply is in charge of the emission module. This supply is based on a low dropout linear regulator which allows a voltage variation between 0.8 V and 3.46 V (see Figure 4). This power variation lets the board modify its emission range. The regulator is connected to a digital SPI potentiometer which varies the load of the ADJ pin modifying the output of the source. Thanks to this digital variable resistor the emission range and power consumption can be software controlled.

*R*102 and *R*114 are 15 *K*Ω resistors, and potentiometer *D*53 modifies its value from 0 Ω to 100 *K*Ω with an 8 bits SPI frame, so 256 levels of approximately 390 Ω are managed. Resistors *R*102 + *D*53 and *R*114 form the resistor divider network necessary to set the output voltage. With this configuration, *V_emis_* follows Equation 1:

(1) $$V_{\text{emis}}=V_{\text{adj}}\frac{(R102+D53)+R114}{R114}$$

where *V_adj_* has a nominal voltage of 0.4 V. *V_emis_* minimum value of 0.8 V is achieved for *D*53 = 0Ω, and maximum value of 3.46 V for *D*53 = 100*K*Ω

The second power supply is in charge of the rest of the electronics including the microcontroller. Analog and digital lines, both of 3.3 V, are separated and short circuited just in one point to reduce noise.

The power consumption of the board depends on the settings of the emission power supply. Table 1 shows the consumption characteristics of the board for different values of the adjustable power supply during a 50% duty cycle of the emission signal.

### 2.2. Emission Module

**Figure 5.**
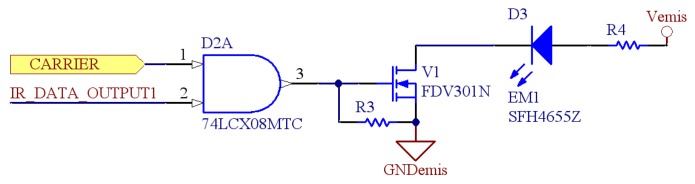
Schematic of one emission module.

The emission module is composed of 12 different emitters. Each sensor set is composed of a narrow beam infrared led and logic gates to create the modulation as the one shown in Figure 5. The infrared leds have their nominal half intensity angle at *±*20°, a 100 mA forward current, a maximum power consumption of 180mW and a nominal switching time of 12ns.

**Figure 6.**
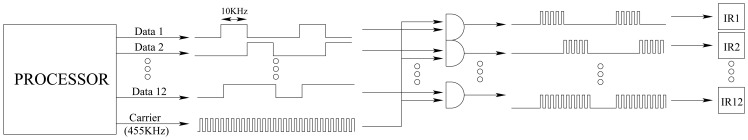
Emission module diagram.

Communication is based on frequency modulation with data at 10 KHz over a carrier of 455 KHz (see Figure 6 for more details). Finally, a FET transistor is added to power on the emitter.

The modification of the *V_emis_* power supply changes the current that passes through the emitter modifying the emission range. For a minimum value of 0.8V, a 40 cm range is achieved while the maximum range is approximately 6m for *V_emis_*=3.46 V.

### 2.3. Reception Module

The reception module is divided in two different submodules. A first submodule is in charge of the data reception while the second one takes care of detecting the intensity of the signal. The division in two submodules allows the board to receive data independently of the signal intensity. In the first module, the board is able to work as a simple communication system, where the data are demodulated and received without the extraction of the emitters location. The second submodule measures the intensity of infrared signals during the reception of a frame. To ensure a proper measure of the signal intensity, intensity and demodulating sensors must have the same orientation and are therefore positioned on top of each other.

**Figure 7.**
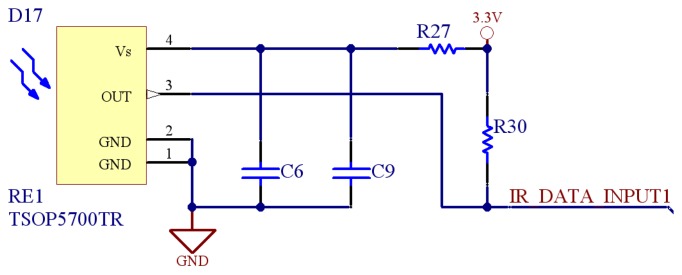
Schematic of one data reception submodule.

The data reception submodule is based on a miniaturized infrared receiver for remote control (see Figure 7). The sensor is packed with a PIN diode and a preamplifier, and the demodulated output signal can directly be decoded by a microprocessor. The signals are received trough digital inputs in the microcontroller.

**Figure 8.**
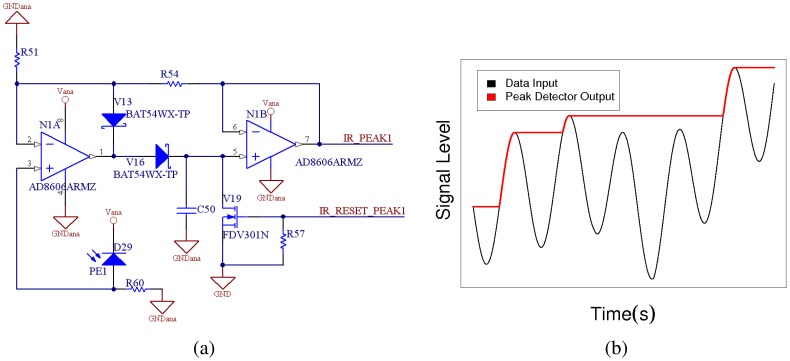
(a) Schematic of one peak detector module. (b) Signal intensity detection.

**Figure 9.**
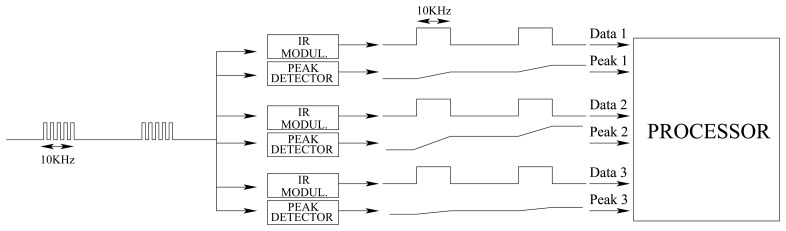
Reception module diagram.

The signal intensity submodule is based on a PIN diode and two operational amplifiers. Figure 8a shows a peak detector system with a *R*54/*R*51 gain. When the photodiode starts receiving infrared signals, the circuit starts charging capacitor *C*50. Once the signal is exhausted, the system keeps the voltage in the capacitor (if no leak currents are taken into account). If a higher strength signal arrives to the diode, it will continue charging the capacitor. If the signal arrived has a lower intensity than the actual value stored in the capacitor, the peak detector will keep its value (see an example in Figure 8b). The outputs from the peak detector face 12 analog to digital converters in the microcontroller. Finally, a hardware reset based on a FET transistor (*V* 19) is added to the circuit for discharging the capacitor. The resets are managed trough 12 independent output pins. The complementary activities of the data and signal intensity reception submodules are sketched in Figure 9.

### 2.4. Communication Module

The communication module has been designed to be the slave of a main processor system. Two buses, I2C and RS232, have been incorporated for facilitating the use of the board.

In the I2C communication, the range and bearing board acts as slave of the main processor system. The board takes care of the requests of transmission and is continuously checking for incoming frames. The main processor system polls continuously the board to check if any communication has been received.

In the serial port communication, interruptions are enabled in both directions. The master board is able to send orders of transmission or range modifications. Once a frame is demodulated by the communication board, it interrupts the master and transmits the demodulated data, the estimated angle and the distance to the emitter.

In both communication types, the master has the control of the emission range. The modification of the power supply output can be ordered at anytime and results in an immediate modification of the emission range.

## 3. Model Description

**Figure 10.**
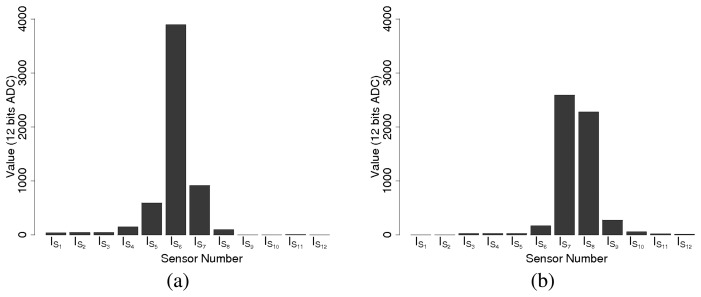
Sensory map for two different reception frames. (a) The emitter is approximately facing a reception sensor. (b) The emitter is in some point in between two reception sensors.

Due to the hardware design, one single transmission is likely to be detected by several infrared sensors. For getting the correct location information, an internal data fusion must be carried out before supplying data to the master board. Figure 10 shows an example of a reception diagram. We observe that several sensors are receiving the same information but there is a signal strength difference between the sensors. In Figure 10a, the emitter is facing sensor *I_S_*_6_ while in Figure 10b the orientation of the emitter is between *I_S8_* and *I_S_*_9_.

For getting a more accurate measure on the bearing we implement a linear combination between the two sensors with highest power signal following Equation 2:

(2) $$\tilde{\xi }=\frac{\phi_{\max_{1}}{\hat{v}}_{\max_{1}}+\phi_{\max_{2}}{\hat{v}}_{\max_{2}}}{{\hat{v}}_{\max_{1}}+{\hat{v}}_{\max_{2}}}$$

where ξ̃ is the estimated angle, *ϕ_max1_* and *ϕ_max2_* are the orientation angles of the two maximum reception value sensors and *V̂_max_*_1_ and *V̂_max_*_2_ are the received values on both sensors.

**Figure 11.**
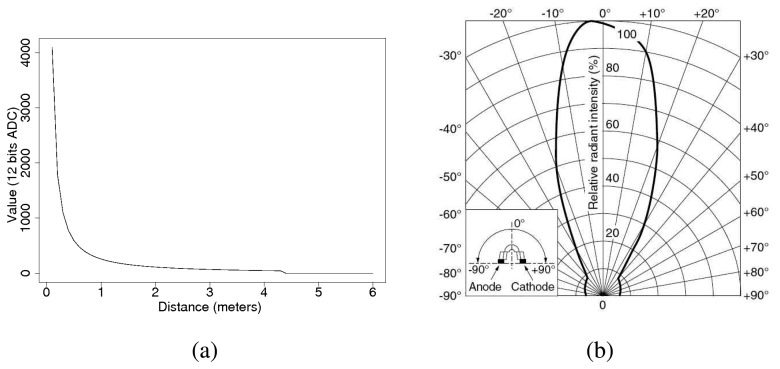
(a) Reception values for different distance transmissions when emitter and receiver sensors are facing each other. (b) Radiation diagram for the PIN diode of the peak detector. (Obtained from the PD100MF0MPx Datasheet).

**Figure 12.**
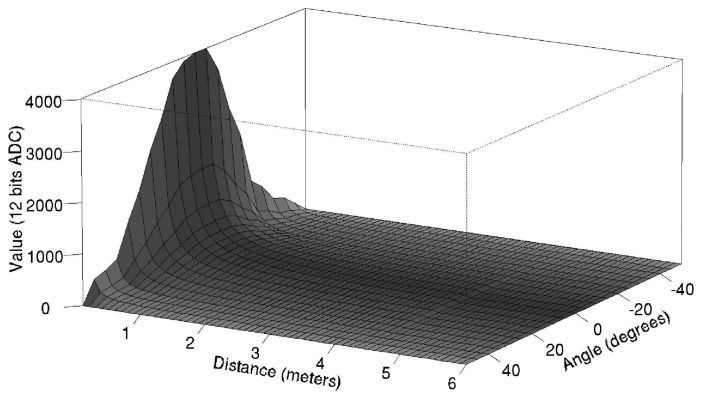
Reception values for different distance and angle transmissions.

It is difficult to rely on a single sensor to determine accurately the distance of the emitter. Therefore, we use a linear combination based on the estimated bearings and distances provided by the two sensors that detected the strongest signal intensity. To this end, we have devised an empiric relationship between the ADC values and the distance when the emitter and receiver sensors are facing each other as shown in Figure 11a. As the receiver sensors have a maximum sensibility angle at −3° which decreases according to Figure 11b, the relationship between the ADC values and the distance must be extended to a 3D graph as shown in Figure 12. Following this graph, we calculate the estimated distances ρ̃*_max_*_1_ and ρ̃*_max_*_2_ from the emitter to each of the two maximum sensors from the received ADC values ν̂*_max_*_1_ and ν̂*_max_*_2_ respectively.

**Figure 13.**
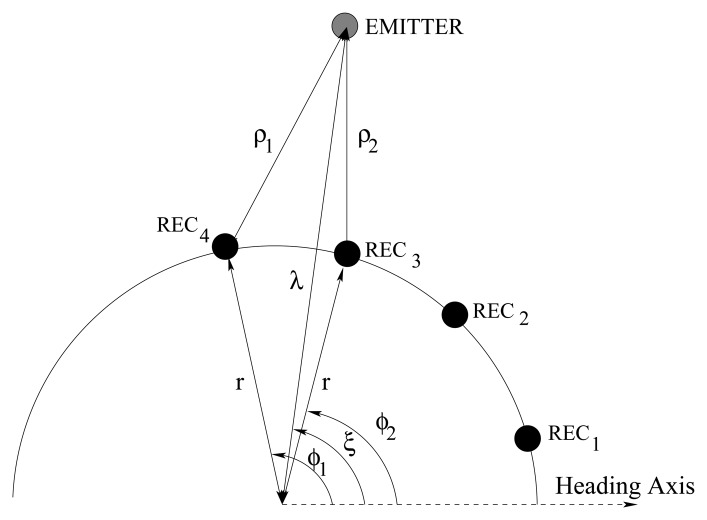
Reception diagram for a specific location and its distance to the different sensors.

Applying the law of cosines, we devise a relationship to calculate two estimated distances *λ_maxi_* from the center of the board to the emitter following Equation 3 (see also Figure 13):

(3) $${\tilde{\lambda }}_{max_{i}}^{2}-2r{\tilde{\lambda }}_{max_{i}}\cos\left(\left|\tilde{\xi }-\phi_{max_{i}}\right|\right)+(r^{2}-{\tilde{\rho }}_{max_{i}}^{2})=0,i=1,2$$

where *r* is the board radius.

We obtain the estimated distance *λ* of the emitter by averaging out *λ_maxi_* as shown in Equation 4:

(4) $$\tilde{\lambda }=\frac{{\tilde{\lambda }}_{\max_{1}}+{\tilde{\lambda }}_{\max_{2}}}{2}$$

## 4. Experimental Evaluation

Two different experiments have been run to characterize and validate the localization and communication system. The first experiment is developed in an obstacle-free environment with 2 nodes, while the second experiment is a multi-node network in a non-obstacle-free environment.

### 4.1. Two nodes in an obstacle-free environment experiment

**Figure 14.**
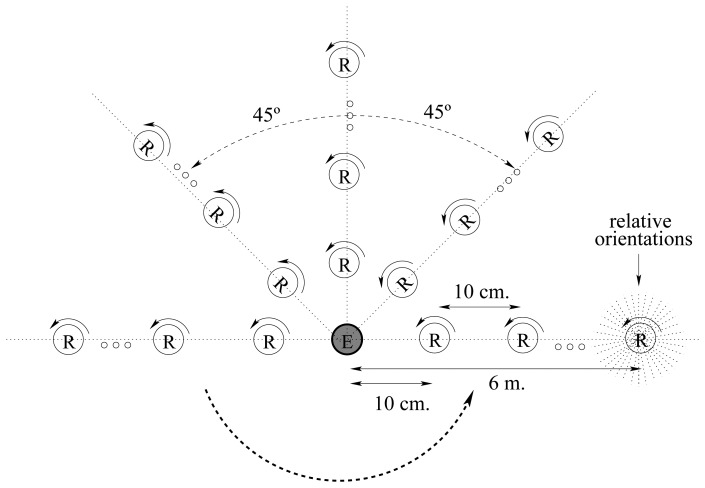
Physical arrangement of the boards for the experiments.

One emitter and one receiver board are placed in an obstacle-free environment from 10 cm to 6 m, in 10 cm intervals as shown in Figure 14. At each distance the board is placed with 8 different orientations (at 45*^°^* intervals) from the emitter to avoid bias on the emitter transmission. The emitter stays in place while the receiver is rotated at 10*^°^* intervals in each position. At each position, the receiver waits till 100 messages are received and stores 12 signal intensity values. We have repeated this test for 60 positions along each of 8 directions and 36 relative orientations for 10 different boards.

**Figure 15.**
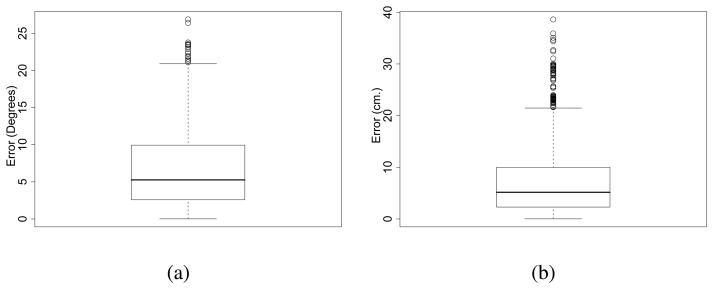
Algorithm performance for calculating (a) the angle and (b) the distance error. Each box comprises observations ranging from the first to the third quartile. The median is indicated by a horizontal bar, dividing the box into the upper and lower part. The whiskers extend to the farthest data points that are within 1.5 times the interquartile range. Outliers are shown as circles.

**Figure 16.**
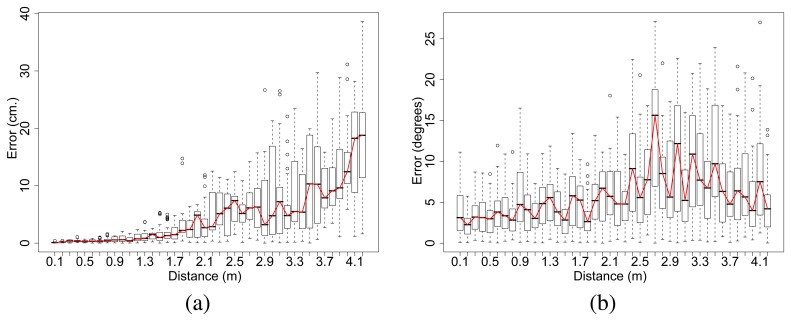
(a) Standard deviation of the distance estimated with respect to the actual distance between emitter and receiver. (b) Standard deviation of the angle estimated with respect to the actual relative orientation between emitter and receiver.

The error for each range and bearing of the receiving board over all measures was calculated. The error on the bearing was 6.69*^°^* on average and 26.87*^°^* in the worst case. The error on the range was 7.18 cm on average and 38.62 cm in the worst case (see Figure 15 for the error distribution). The variation of the distance and angle error with respect to the range are shown in Figure 16. We observe errors of 1 cm on average for distances of 1 m and errors of 20 cm on average for distances of 4 m. Standard deviation across all the ranges is 5% of the distance for the range and *±*10*^°^* for bearing in the worst case.

**Figure 17.**
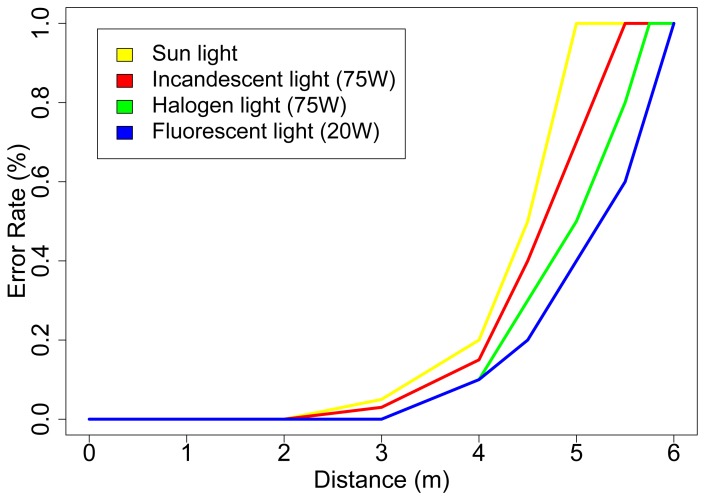
Error rate of the communication system.

Different light conditions introduce divers infrared component signals. As mentioned in Section 2. the board implements a signal noise correction mechanism based on the ambient light conditions. The board keeps continuously measuring the infrared ambient light and subtracts it from the measure when a signal is received. This mechanism allows the board to be moved from one place to another with different light conditions. Depending on the amount of infrared light in the environment, the range of the board is slightly reduced. We have tested the board in four different light conditions environments: *i)* sun light, *ii)* incandescent light with a 75 W lamp, *iii)* halogen light with a 75 W lamp and *iv)* fluorescent light with an energy-saving lamp of 20 W. Figure 17 shows the error rate of the communication system.

Notice that for distances greater than 4 meters (in a fluorescent light environment) the board receives the data but, due to the sensibility of the photodiode sensors, it is not able to estimate the distance to the emitter. Instead, it returns a tag informing that the emitter is too far, the data and a bearing estimate calculated using the position of the data receivers which have demodulated a correct frame.

### 4.2. A multi-node network in a non-obstacle-free environment experiment

**Figure 18.**
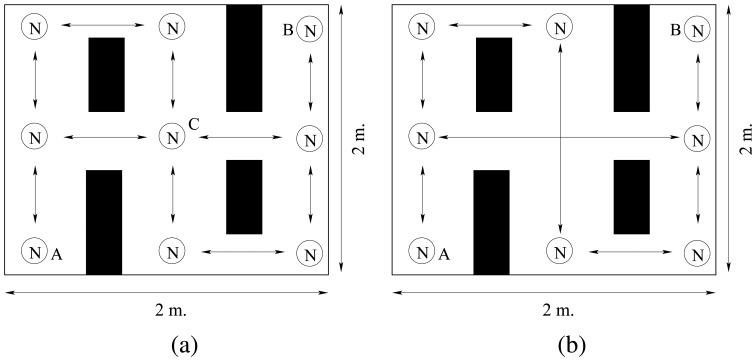
(a) Physical arrangement of a multi-node network on an environment with obstacles experiment. (b) Physical arrangement of the network when *node C* has been removed. Communication axis are represented by a black double arrow.

The network is composed of 9 nodes in a 2×2 m^2^ arena with 4 different obstacles as shown in Figure 18a.

*Node* A is offering a measure (e.g. temperature) to the network needed by *node B*, which also requires information about the location of *node A*. Each node in the network stores the information offered by *node A* and its relative position. A node which has received information about *node A* broadcasts the data received and the relative location of *node A*, that will be received by other nodes in the network, and so on.

**Figure 19.**
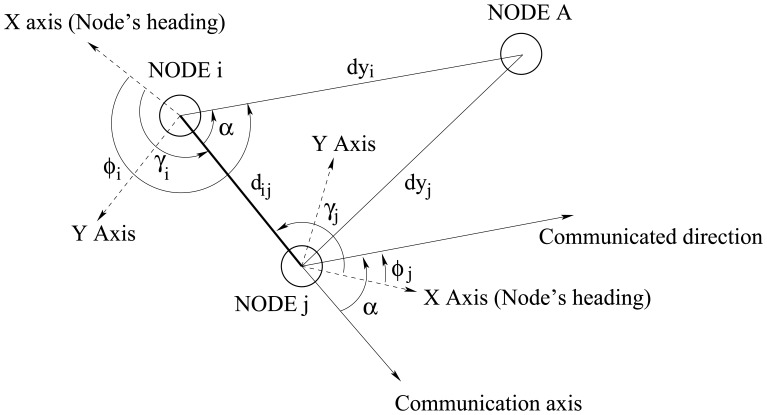
Nodes sharing information about the estimated location of node A.

Since the nodes do not have an absolute reference system, they must rely on a common reference axis, the communication axis. Figure 19 shows how information about the estimated location of *node A* is transmitted from *node i* to *node j*. In a first step, *node i* transmits its estimate of the distance *dy_i_* and direction *ϕ_i_* of *node A* to *node j*. For the direction, the value transmitted is the angle *α*, obtained from (*x003D5*)*_i_* using the communication beam as reference axis: *α* = *ϕ_i_* − *γ_i_*. In a second step, *node j* transforms the received data into its own coordinates system. First, it calculates the direction pointed by *node i* as *ϕ*_j_ = *γ_j_* + *α* − *π*. Then, *nodej* calculates the location *loc_j_* = (*x, y*) *of node A* related to its own reference frame using *node i* information and the simple trigonometric equations *x* = *d_ij_cosγ_j_* + *dy_i_cosϕ_j_* and *y* = *d_ij_sinγj* + *dy_i_sinϕ_j_*.

Due to the physical characteristics of the infrared signals, receivers must have direct vision to the emitters to receive messages. In the example shown, nodes receive information from different paths which is averaged with data from previous communication. Once the different nodes receive information about the data and location of *node A* they broadcast the information. In the middle of the experiment, we remove *node C* from the network (see Figure 18b) and observe how it affects the location information.

**Figure 20.**
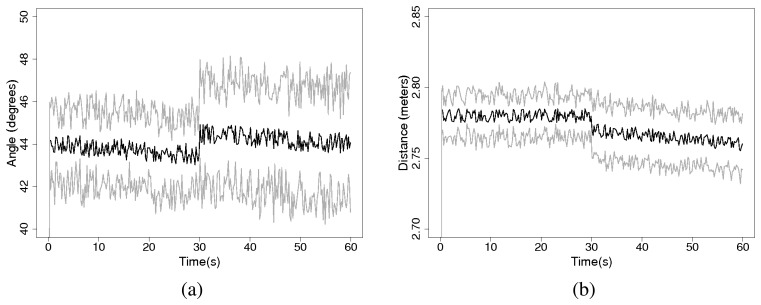
(a) Average bearing estimate (black) and standard deviation (grey) for the multinode experiment over 30 replications. (b) Average range estimate (black) and standard deviation (grey) for the multi-node experiment over 30 replications.

Experiments last for 60 seconds and there are 30 replications. We obtain the data and location information of *node A* stored in *node B* each 100 ms. Results show that data information is always received by node *B* with a maximum error of 5*^°^* for the bearing and of 8cm for the distance. Once a node is removed from the network, the relative position estimate of *node A* suffers a small change because of the removed node. This is a consequence of the increased transmission distance between the nodes. Figure 20 shows the mean and standard deviation for the range and the bearing over the 60 seconds experiment.

## 5. Conclusions

In this paper we have described the design of an open board for localization and local communication.

The system provides a communication rate up to 5kbps with a frequency modulation which allows robustness to light conditions. The range of communication can be modified from 0 to 6 meters by software and in real time. The system gets the data and extracts range and bearing from the communication at the same time. The board operates with a maximum error of 1 cm in range and 2*^°^* in bearing at dis-tances below one meter. For longer distances, performance degrades gracefully with a maximum error of 38.62 cm in range and 26.87*^°^* in bearing at 6 meters.

Although the board has been designed for robotics tasks, it can easily be used in different applications such as smart sensors, intelligent ambients, home automation, and so on. Thanks to the open hardware license under which the board is released, along with the full documentation and the low cost of production, this board provides researchers with a new and versatile communication tool for systems made of multiple interacting entities.

Potentials for further improvements to the system have been explored. Increasing the number of peak reception modules will offer a better spatial information which will reduce the range and bearing error. In the near future, we plan to extend the range and bearing board to work in 3D spaces. This implementation will allow the nodes to be located in different planes and to create a 3D spatial map. It will give to the network the capability of having nodes which could move freely in the space keeping them localized. For the accomplishment of the 3D extension, a media control access will be implemented on top of the existing firmware.

## Figures and Tables

**Table 1.** Consumption characteristics of the board.

*V_emis_*	Min (mW)	Nominal(mW)	Max(mW)
0%	70	74	80
10% (0.8 V)	110	114	119
50% (2.13 V)	392	396	400
100% (3.46 V)	674	679	683

## Footnotes

* For an exhaustive description of the board see http://www.rbz.es/randb/.
